# Unique Variation of Superficial and Deep Palmar Arches: A Case Report With Literature Review

**DOI:** 10.7759/cureus.78792

**Published:** 2025-02-09

**Authors:** Punnapa Raviteja, Mrudula Chandrupatla, Ariyanachi K

**Affiliations:** 1 Department of Anatomy, All India Institute of Medical Sciences, Bibinagar, Hyderabad, IND

**Keywords:** deep palmar arch, dorsal metacarpal artery, radial artery, superficial palmar arch, ulnar artery

## Abstract

The design and structure of the palmar vascular arches and their variations constitute some of the most intriguing and difficult areas of anatomy. Additionally, cardiac revascularisation is now being accomplished using radial artery harvesting, which necessitates collateral circulation in the hand through the palmar arches. In this case report, we outline a unique pattern of the superficial palmar arch (SPA) and deep palmar arch (DPA) in terms of their formation and branching pattern. The first and second dorsal metacarpal arteries (DMAs) emerged from the radial artery. Both the first and second DMAs participate in the formation of SPA and DPA, respectively. Additionally, there was an anastomosis between the third common digital artery (CDA) and the proper digital artery, which has not yet been documented in any literature, and the third CDA was derived from the DPA. Understanding the variations in the arteries of the hand is becoming increasingly crucial for surgeons due to recent advancements in microsurgical techniques for vascular repair.

## Introduction

The lumbricals are supplied by the branches of the superficial palmar arches (SPA) and deep palmar arches (DPA), whereas the interossei are supplied by the dorsal metacarpal arteries (DMAs) and the DPA [1]. Both the lumbrical muscle flap and the pedicled interossei are used in hand reconstructive surgery [2,3]. The hand's vasculature is greatly impacted by inadequate palmar arches, which are rather prevalent when insufficient collateral circulation exists. Here, we present a unique case of unilateral variation in the formation and branching pattern of SPA and DPA with variant termination of the radial artery, which emphasises the importance of the Allen test, ultrasonography, or angiography to check for patency of collateral circulation prior to undertaking standard procedures such as arterial blood gas (ABG) collection and microsurgical operations like arterial graft and hand reconstruction surgeries [4].

## Case presentation

**Figure 1:**
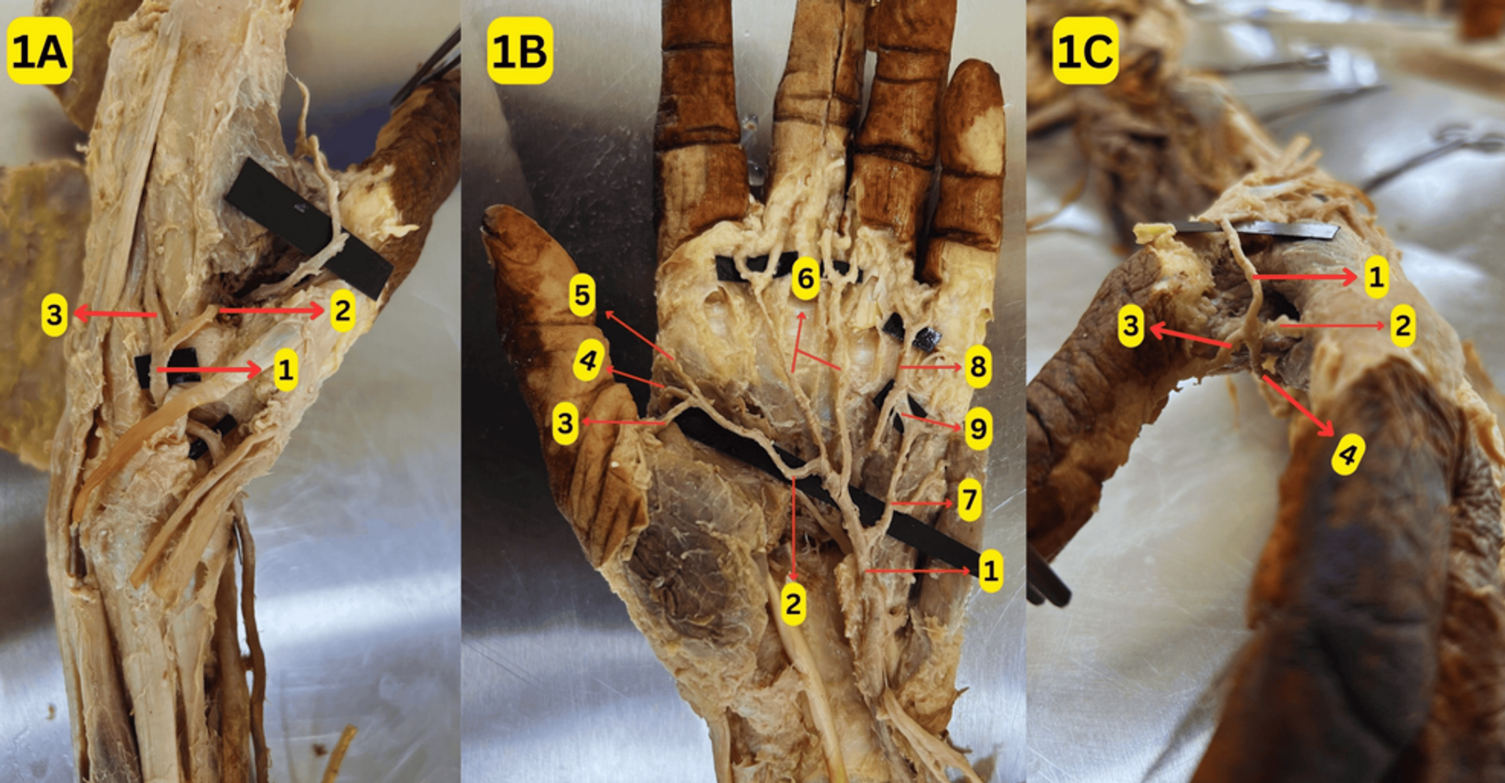
Variation in the formation and branches of superficial palmar arches (SPA) 1A: Radial artery termination: (1) radial artery; (2) first dorsal metacarpal artery; (3) second dorsal metacarpal artery 1B: SPA and its branches: (1) superficial branch of the radial artery; (2) superficial palmar arch; (3) princeps pollicis artery; (4) the first dorsal metacarpal artery contributes to the formation of the superficial palmar arch; (5) radialis indices artery; (6) two common digital arteries; (7) proper digital artery; (8) the third common digital artery arising from the deep palmar arch; (9) anastomosis between the third common digital and proper digital arteries 1C: The first dorsal metacarpal artery contributes to the formation of the SPA: (1) first dorsal metacarpal artery; (2) radialis indices artery; (3) princeps pollicis artery; (4) the first dorsal metacarpal artery contributes to the formation of the superficial palmar arch Original image by the authors

During undergraduate and postgraduate practical sessions on the palm of a donated 61-year-old female cadaver, we observed a unilateral variation in the formation and branching pattern of SPA and DPA, along with the variation in the termination of the radial artery. After crossing the anatomical snuff box in the dorsum of the hand, the radial artery bifurcated into two branches: first and second DMAs, as shown in Figure 1A. SPA was formed by the anastomosis between the superficial branch of the ulnar artery and the first DMA in the first interosseous space, as shown in Figures 1B, 1C. The princeps pollicis and radialis indices, two common digital arteries (supplying the contiguous sides of the index and middle fingers and the contiguous sides of the middle and ring fingers) and one proper digital artery (supplying the ulnar side of the little finger) were seen arising from this arch, as shown in Figure 1B.

**Figure 2:**
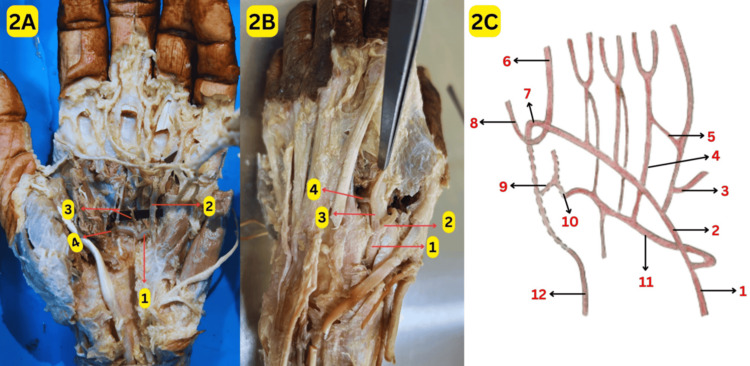
Variation in the formation and branches of deep palmar arches (DPA) 2A: Branches of the DPA: (1) deep palmar arch; (2) third common digital artery; (3) first and second palmar metacarpal arteries; (4) perforating artery contributing to the formation of the deep palmar arch in the second interosseous space 2B: Perforating artery of the second metacarpal artery contributing to the formation of the DPA: (1) radial artery; (2) first dorsal metacarpal artery; (3) second dorsal metacarpal artery; (4) perforating artery 2C: Schematic representation of variations in palmar arches: (1) ulnar artery; (2) superficial palmar arch; (3) branch to the ulnar side of the little finger; (4) third common digital artery from the deep palmar arch; (5) anastomosis between third common digital and proper digital arteries; (6) radialis indices artery; (7) first dorsal metacarpal artery contributing to the formation of the superficial palmar arch; (8) princeps pollicis artery; (9) second dorsal metacarpal artery; (10) perforating artery contributing to the formation of the deep palmar arch; (11) deep palmar arch; (12) radial artery Original image by the authors

The DPA was formed by an anastomosis between the second DMA via the perforating artery and the deep branch of the ulnar artery in the second interosseous space, as shown in Figures 2A, 2B. The first and second palmar metacarpal arteries and the third common digital artery (CDA) supplying the contiguous sides of the ring and little fingers originated from this arch, as shown in Figure 2B. In addition, there was also an anastomosis between the third CDA and the proper digital artery, as shown in Figure 1B. The schematic representation of these variations is depicted in Figure 2C.

## Discussion

Several researchers have proposed classifications for the various SPA branching patterns and formations. Based on the key arteries that contribute, Adachi categorised the SPA into three primary groups: the ulnar type (where the contribution is only from the ulnar artery), the radial-ulnar type (both the radial and ulnar arteries contribute to the formation of the SPA), and the median-ulnar type (the median artery forms a significant part of the SPA) [5]. According to Huber, there are two main categories of SPA: full arch, which exhibits anastomosis, and incomplete arch, which does not exhibit anastomosis [6]. Coleman and Anson [7] provided the first thorough categorisation of SPA, which is explained in detail in Table 1. Based on these classifications, our finding is type E, but in the present case, SPA is completed by the first DMA in the first interosseous space. However, Gnanasekaran and Veeramani reported a similar conclusion to ours and, based on their observation in 55 hands [8], established a new categorisation, which is explained in detail in Table 1. According to this classification, the princeps pollicis immediately emerges from the first web arch, feeding both sides of the thumb, and has characteristics of both types 2 and 3.

**Table 1:** Classification of superficial palmar arch (SPA) patterns

Authors	Classification
Adachi (1928) [5]	Ulnar type (SPA is formed by the ulnar artery alone), radial-ulnar type (both radial and ulnar arteries contribute to the formation of the SPA), and median-ulnar type (the median artery forms an important component of the SPA).
Huber (1930) [6]	complete arch (contributing vessels anastomose with each other); incomplete arch (contributing vessels do not anastomose with each other).
Coleman and Anson (1961) [7]	Type A: classical type formed by contributions from the radial and ulnar arteries—the superficial palmar branch of the radial artery and the larger ulnar artery. Type B: SPA is formed entirely by the ulnar artery alone. Type C: ulnar artery with an enlarged median artery. Type D: the arch is formed by the combination of three vessels: radial, median, and ulnar. Type E: the arch is formed by the ulnar artery and completed by a large-sized vessel derived from the deep arch.
Gnanasekaran and Veeramani (2019) [8]	Type 1: the terminal branch of the ulnar arteries anastomose with the superficial branch of the radial artery. Type 2: the terminal branch of the ulnar arteries anastomose with the first dorsal metacarpal artery in the first web space. Type 3: the terminal branch of the ulnar arteries anastomose with both the superficial branch of the radial artery and the first dorsal metacarpal artery in the first web space. Type 4: the terminal branch of the ulnar arteries anastomose with the first palmar metacarpal artery.

Table 2 details the commonly followed classification of DPA formation [9-11]. Another interesting finding in the present case is that DPA does not fit into any of the classifications described above. Here, it is formed by a deep branch of the ulnar artery joining with the second DMA through a perforating artery in the second interosseous space.

**Table 2:** Classification of deep palmar arch (DPA) patterns

Authors	Classification
Ruengsakulrach et al. (2001) [10]	Complete DPA: The terminal portion of the deep palmar branch of the radial artery had a connection with the deep palmar branch of the ulnar artery. Incomplete DPA: The deep palmar branch of the radial artery did not have any connection with the ulnar artery at the deep palmar level.
Loukas et al.​​​​​​​ (2005) [9]	Type 1: The DPA is formed by the deep volar branch of the radial artery and the inferior deep branch of the ulnar branch. Type 2: The DPA is formed by the deep volar branch of the radial artery and the superior deep branch of the ulnar artery. Type 3: The DPA is formed by the deep volar branch of the radial artery with both deep branches of the ulnar artery.
Dawani et al. (2022) [11]	Type 1: The radial artery unites with the deep branch of the ulnar artery. Type 2: The second dorsal metacarpal artery, which branched from the radial artery, communicated with the DPA through a perforating artery, and this perforating artery completed the arch.

A similar finding was reported in 3.3% of the observed specimens in a study by Dawani et al., who proposed a new classification [11]. This similar variation was identified by Coleman and Anson as a kind of incomplete DPA [7]. Olave and Prates documented that the DPA in 13.3% of the hands was formed by the radial artery passing through the second interosseous space [12]. Ikeda et al. also described a case where the radial artery that passed through the first interosseous space was present, but the DPA was mostly caused by the perforating artery of the second interosseous space [13]. However, in the present case, the radial artery terminated in the dorsum of the hand, bifurcating into the first and second DMAs, and the first DMA completed the superficial arch in the first interosseous space.

Contrary to the description of the branches of SPA and DPA given in standard anatomical textbooks, the third CDA supplying the contiguous sides of the ring and little fingers originated from the DPA, and there was also anastomosis between the third CDA and the proper digital artery, which is not documented in the literature. Blood islands are formed by mesenchymal angioblastic tissues, which are then hollowed down and lined by squamous endothelial cells. These discrete areas come together to create the vascular plexus, from which some may develop these variants or degenerate. According to Arey, the distinct route selection of the primitive vascular plexus may lead to blood vessel abnormalities. These may include the preservation of vessels that should be destroyed, the loss of vessels that are normally maintained, insufficient growth, and the blending and absorption of typically distinct portions [14].

## Conclusions

Although intricate anatomical variations of the hand have been documented in several studies, the lack of specificity in identifying collateral circulation differences, such as the coexistence of an incomplete DPA and an incomplete SPA in the same hand, which might influence the decision to remove the radial artery, limits the applicability of these studies. Therefore, it is essential for a surgeon to recognise the functional arterial arch before performing any intervention.
